# Developing reduced SNP assays from whole‐genome sequence data to estimate introgression in an organism with complex genetic patterns, the Iberian honeybee (*Apis mellifera iberiensis*)

**DOI:** 10.1111/eva.12623

**Published:** 2018-03-30

**Authors:** Dora Henriques, Melanie Parejo, Alain Vignal, David Wragg, Andreas Wallberg, Matthew T. Webster, M. Alice Pinto

**Affiliations:** ^1^ Mountain Research Centre (CIMO) Polytechnic Institute of Bragança Bragança Portugal; ^2^ Centre of Molecular and Environmental Biology (CBMA) University of Minho Braga Portugal; ^3^ Agroscope Swiss Bee Research Centre Bern Switzerland; ^4^ Institute of Bee Health Vetsuisse Faculty University of Bern Bern Switzerland; ^5^ GenPhySE Université de Toulouse INRA INPT INP‐ENVT Castanet Tolosan France; ^6^ The Roslin Institute University of Edinburgh Edinburgh UK; ^7^ Department of Medical Biochemistry and Microbiology Science for Life Laboratory Uppsala University Uppsala Sweden

**Keywords:** *Apis mellifera iberiensis*, fixation index, informative SNPs, reduced SNP assays

## Abstract

The most important managed pollinator, the honeybee (*Apis mellifera* L.), has been subject to a growing number of threats. In western Europe, one such threat is large‐scale introductions of commercial strains (C‐lineage ancestry), which is leading to introgressive hybridization and even the local extinction of native honeybee populations (M‐lineage ancestry). Here, we developed reduced assays of highly informative SNPs from 176 whole genomes to estimate C‐lineage introgression in the most diverse and evolutionarily complex subspecies in Europe, the Iberian honeybee (*Apis mellifera iberiensis*). We started by evaluating the effects of sample size and sampling a geographically restricted area on the number of highly informative SNPs. We demonstrated that a bias in the number of fixed SNPs (F_ST_ = 1) is introduced when the sample size is small (*N* ≤ 10) and when sampling only captures a small fraction of a population's genetic diversity. These results underscore the importance of having a representative sample when developing reliable reduced SNP assays for organisms with complex genetic patterns. We used a training data set to design four independent SNP assays selected from pairwise F_ST_ between the Iberian and C‐lineage honeybees. The designed assays, which were validated in holdout and simulated hybrid data sets, proved to be highly accurate and can be readily used for monitoring populations not only in the native range of *A. m. iberiensis* in Iberia but also in the introduced range in the Balearic islands, Macaronesia and South America, in a time‐ and cost‐effective manner. While our approach used the Iberian honeybee as model system, it has a high value in a wide range of scenarios for the monitoring and conservation of potentially hybridized domestic and wildlife populations.

## 1 INTRODUCTION

Biodiversity, including the genetic diversity within and between populations, is a unique heritage whose conservation is imperative for the benefit of future generations (Frankham, Ballou, & Briscoe, 2002). This is particularly important for organisms like the honeybee (*Apis mellifera* L.), which, through the pollination service it provides, plays a critical role in ecosystem functioning and in food production for humanity. The honeybee is under pressure worldwide due to multiple factors, ranging from emergent parasites and pathogens, and the overuse of agrochemicals, to the less publicized introgressive hybridization mediated by human management (reviewed by Potts et al., 2010; van Engelsdorp & Meixner, 2010). In a global world, where the circulation of commercial queens and package honeybees occurs at a rapid pace, and at large scale, reliable tools for monitoring genetic diversity are becoming indispensable.

The honeybee exhibits high diversity, with 31 currently recognized subspecies (Chen et al., 2016; Engel, 1999; Meixner, Leta, Koeniger, & Fuchs, 2011; Sheppard & Meixner, 2003) belonging to four main evolutionary lineages (western and northern Europe, M; south‐eastern Europe, C; Africa, A; Middle East and Central Asia, O). Of the 31 subspecies, the Iberian honeybee *A. m. iberiensis* (M‐lineage) has received the most attention with numerous genetic surveys (Chávez‐Galarza et al., 2015; and references therein). These have consistently shown the existence of a highly diverse and structured subspecies defined by two major clusters forming a sharp cline that bisects Iberia along a north‐eastern–south‐western axis (Arias, Rinderer, & Sheppard, 2006; Chávez‐Galarza et al., 2017; Smith et al., 1991). Such complexity has been shaped by recurrent cycles of interacting selective and demographic processes, typical of long‐term glacial refugia organisms (Chávez‐Galarza et al., 2013, 2015, 2017). However, this genetic legacy might be at risk if Iberian beekeepers adopt a strategy of importing commercial strains belonging to the highly divergent lineage C, as is occurring at large‐scale throughout western and northern Europe north of the Pyrenees. Since the early 20th century, beekeeping activity in this part of Europe has been characterized by colony importations and queen breeding with mostly C‐lineage honeybees De la Rúa, Jaffé, Dall'Olio, Muñoz, & Serrano, 2009); which are known for their docile nature and high productivity (Ruttner, 1988). This human‐mediated gene flow has threatened *A. m. mellifera,* the other M‐lineage subspecies besides *A. m. iberiensis* in Europe. Indeed, the genetic integrity of *A. m. mellifera* has been compromised by introgressive hybridization and, in some areas, it has even been replaced by subspecies of C‐lineage ancestry (Jensen, Palmer, Boomsma, & Pedersen, 2005; Pinto et al., 2014; Soland‐Reckeweg, Heckel, Neumann, Fluri, & Excoffier, 2009). Yet, maintaining locally adapted subspecies is crucial for the long‐term sustainability of *A. mellifera* (De la Rúa et al., 2013; van Engelsdorp & Meixner, 2010). Reciprocal translocation experiments have recently shown that local honeybees have longer survivorship (Büchler et al., 2014) and lower pathogen loads (Francis et al., 2014) than introduced ones, reinforcing the importance of preserving the genetic diversity of locally adapted subspecies. Furthermore, it has been advocated that apiculture and commercial breeding could compromise honeybee health by interfering with natural selection (Meixner et al., 2010; Neumann & Blacquière, 2017).

The idea that long‐term sustainability of honeybee populations can only be achieved by preserving natural genetic diversity and coevolved gene complexes has led to the establishment of conservation programmes and protected areas throughout Europe (De la Rúa et al., 2009). To foster and monitor such conservation efforts, reliable, cost‐ and time‐effective tools are needed to accurately assess admixture levels between introduced and native honeybees. For the endangered *A. m. mellifera*, reduced assays of highly informative SNPs have already been developed to estimate C‐lineage introgression (Muñoz et al., 2015; Parejo et al., 2016). However, equivalent tools for application in conservation and breeding efforts are still required for its sister subspecies, *A. m. iberiensis*.

Following the last glacial maximum, honeybees dispersed from the Iberian refugium to colonize a broad territory, extending from the Pyrenees to the Urals (Franck, Garnery, Solignac, & Cornuet, 1998; Ruttner, 1988). This important Iberian reservoir of genetic diversity has not yet been seriously threatened by C‐lineage introgression (Chávez‐Galarza et al., 2015, 2017; Miguel, Iriondo, Garnery, Sheppard, & Estonba, 2007), although this scenario might change as many young beekeepers are attracted by the advertised benefits of commercial strains—being more prolific and docile. In many islands of the Baleares and Macaronesia, for example where the Iberian honeybee was presumably introduced in historical times, the contemporaneous large‐scale importation of commercial C‐lineage queens has resulted in high levels of introgression into the local populations (De la Rúa, Galián, Serrano, & Moritz, 2001, 2003; Miguel et al., 2015; Muñoz, Pinto, & De la Rúa, 2014). The conservation of *A. m. iberiensis* diversity is therefore a priority, especially in the light of climate change as this subspecies is well adapted to a broad range of environments, including hot and dry summer months with limited nectar flows. These adaptations could be a basis for selection of new development cycles suited to new environmental conditions (Le Conte & Navajas, 2008).

A diverse array of molecular tools has been employed to monitor C‐lineage introgression including PCR‐RFLP of the intergenic tRNA^leu^‐cox2 mtDNA region (Bertrand et al., 2015), microsatellites (Jensen et al., 2005; Soland‐Reckeweg et al., 2009) and, more recently, SNPs (Parejo et al., 2016; Pinto et al., 2014). Among these, SNPs are becoming the tool of choice for many applications because they are easily transferred between laboratories, have low genotyping error, provide high‐quality data, are suitable for automation in high‐throughput technologies (Vignal, Milan, SanCristobal, & Eggen, 2002), and are more powerful for estimating introgression in honeybees (Muñoz et al., 2017).

High‐throughput sequencing of whole genomes generates millions of SNPs. Yet, this volume of data is inappropriate for routine conservation purposes, such as breeding and population monitoring. Therefore, the mining of highly informative SNPs from such high genomic resolution data sets is a common approach for developing reduced SNP assays capable of reliable ancestry estimation (Amirisetty, Khurana Hershey, & Baye, 2012; Judge, Kelleher, Kearney, Sleator, & Berry, 2017). While different metrics and approaches (e.g., Delta, I_n_, PCA, outlier tests) can be used for ranking SNPs by information content, the fixation index (*F*_ST_) has been the metric of choice perhaps due to its power (Ding et al., 2011; Karlsson, Moen, Lien, Glover, & Hindar, 2011; Wilkinson et al., 2011), especially when comparing only two highly divergent populations (Hulsegge et al., 2013). Furthermore, some metrics are correlated regarding information content, in particular those based on allele frequencies (Ding et al., 2011; Wilkinson et al., 2011).

In this study, we developed cost‐effective reduced SNP assays from 176 whole‐genome sequences. When developing such tools, to assure that they are accurate and reliable, the diversity and population complexity needs to be considered. Therefore, taking advantage of the large and comprehensive whole‐genome data set for *A. m. iberiensis* (*N* = 117), we first tested the effect of sample size and sampling a geographically restricted area on detecting fixed SNPs. Next, we designed the reduced SNP assays using a training data set to identify highly informative SNPs (*F*_ST_ = 1), which were then validated in holdout and simulated data sets. The constructed SNP assays were revealed to be very powerful for accurately estimating C‐lineage introgression and can thus be applied to support conservation efforts in the Iberian honeybee.

## 2 MATERIALS AND METHODS

### 2.1 Samples

**Figure 1:**
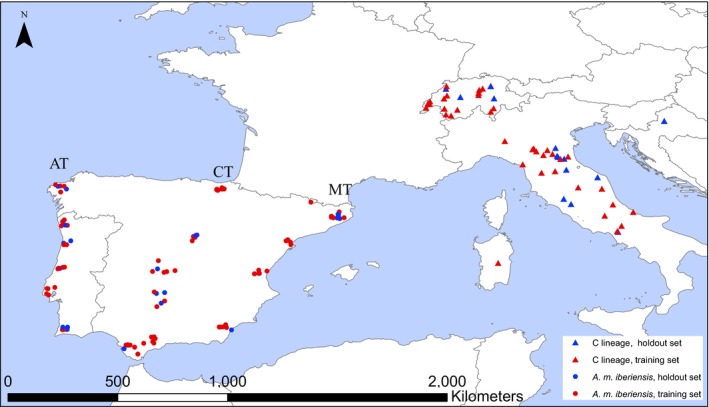
Geographic locations of the 176 whole‐genome sequenced individuals. The Iberian honeybees are distributed across the three transects: Atlantic (AT;* N* = 31), Central (CT;* N* = 61) and Mediterranean (MT,* N* = 25). Each dot represents a single colony and apiary

The whole‐genome sequences used in this study were obtained from 176 pure haploid males, representing 117 *A. m. iberiensis*, 28 *A. m. carnica* and 31 *A. m. ligustica* (DH and MAP, unpublished data; Parejo et al., 2016) sampled across a wide geographical range (Figure 1). All samples were sequenced on an Illumina HiSeq 2500 with an aimed sequencing depth of 10× per individual. Mapping and variant calling were performed following best practices (see Supporting Information for details).

To assess subspecies ancestry and purity of all individuals included in the initial whole‐genome data set (see Supporting Information for details), we inferred model‐based admixture proportions (Q‐values) for *K* = 1 to 5 clusters with 10,000 iterations using the software ADMIXTURE v1.3.0 (Alexander, Novembre, & Lange, 2009). We employed Q‐value thresholds of >0.95 and <0.05 for defining subspecies ancestry and purity of C‐lineage and M‐lineage subspecies, respectively (detailed information in Supporting Information). Convergence between independent runs was monitored by comparing the resulting log‐likelihood scores (LLS) using the default termination criterion set to stop when LLS increases by less than 0.0001 between runs. The optimal number of K clusters was determined using cross‐validation (CV) error as implemented in ADMIXTURE. Q‐values were visualized in R (R Core Team, 2016). To have an overall estimate on population divergence, we calculated in PLINK 1.9 (Chang et al., 2015) the average genomewide pairwise F_ST_ (Weir & Cockerham, 1984) between *A. m. iberiensis, A. m. carnica* and *A. m. ligustica* and between *A. m. iberiensis* and combined *A. m. carnica* with *A. m. ligustica* (C‐lineage).

### 2.2 Effect of sampling bias on the number of fixed SNPs

Starting with a large sample size, which covers a species’ entire geographical range and therefore encompasses its variation, is an important first step for developing SNP assays with high statistical power (Ding et al., 2011; Mariette, Le Corre, Austerlitz, & Kremer, 2002). Using the large (*N* = 117) and geographically comprehensive sample of *A. m. iberiensis* (Figure 1), we assessed the effects of sample size and of sampling a geographically restricted area on the number of fixed SNPs (*F*_ST_ = 1).

**Figure 2:**
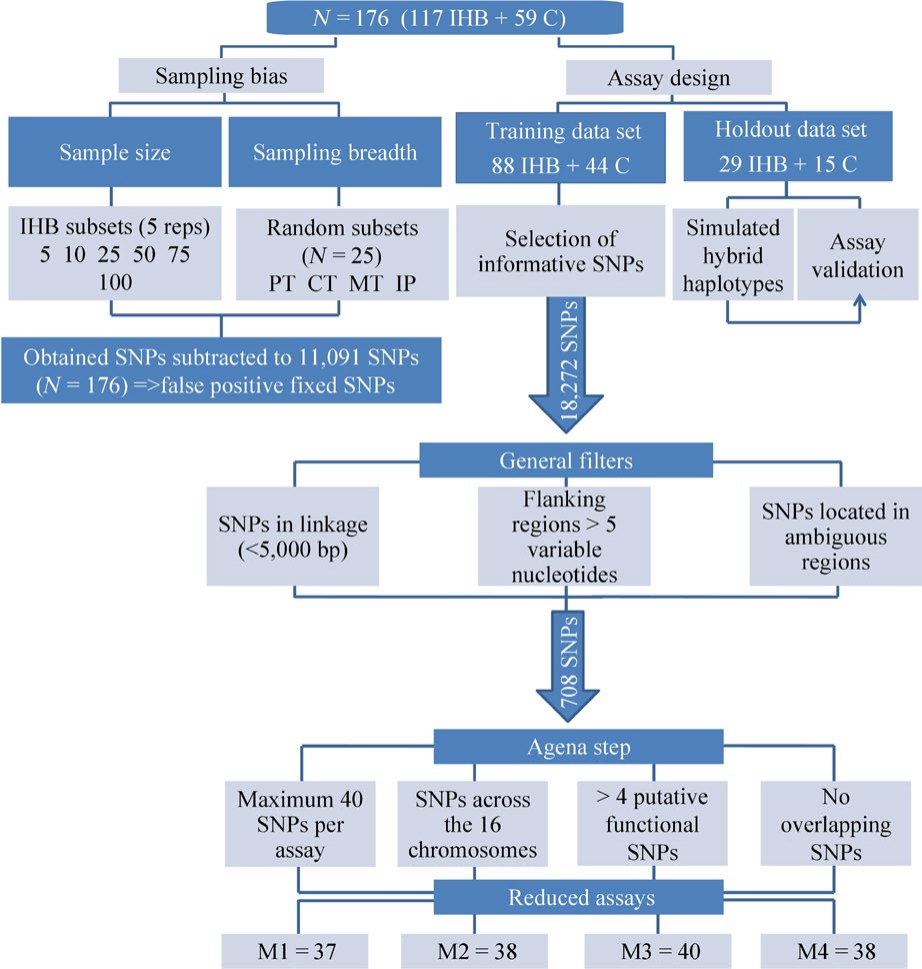
Diagram depicting the different phases of development of the four reduced SNP assays (M1, M2, M3 and M4) using as a baseline whole‐genome sequence data from 117 *Apis mellifera iberiensis* (IHB) and 59 C‐lineage (C)

To test the effect of sample size, we constructed 30 subsets with different sample sizes (*N* = 5, 10, 25, 50, 75 and 100, five replicates each) by randomly choosing individuals from the complete data set (*N* = 117) of *A. m. iberiensis* (Figure 2). Next, we calculated the number of fixed SNPs between each of the 30 *A. m. iberiensis* subsets and the C‐lineage data set (*N* = 59) using PLINK. The number of fixed SNPs identified for each replicate was subtracted from the number of fixed SNPs calculated with the complete *A. m. iberiensis* data set. This approach provided an estimate of the number of SNPs erroneously identified as fixed between the two groups, due to limited sampling effort (false‐positive fixed SNPs).

To test the effect of sampling a geographically restricted area, we constructed four different subsets by randomly choosing 25 individuals (*N* = 25) from the following areas: Portugal (PT; this sample may arise in practice when sampling is country‐limited), Central transect (CT; sampling representing the largest latitudinal distance in Iberia), Mediterranean transect (MT; sampling along the Mediterranean coast mimics the pioneer mtDNA survey carried out by Smith et al., 1991) and across the Iberian Peninsula (IP) to intentionally capturing the entire variation in *A. m*. *iberiensis*. The number of fixed SNPs between the C‐lineage data set (*N* = 59) and each of the four subsets was subtracted from the number of fixed SNPs calculated with the complete *A. m. iberiensis* data set. The number of false‐positive fixed SNPs was then compared among the four subsets (Figure 2).

### 2.3 Assay design

After assessing the effects of sampling bias on the number fixed SNPs, we proceeded with designing the reduced SNP assays for estimating C‐lineage introgression into *A. m. iberiensis* (Figure 2). We followed Anderson's simple training and holdout method to minimize the bias which is introduced when selection and assessment of informative SNPs are based on the same individuals (Anderson, 2010). Accordingly, we set aside a holdout data set, consisting of 29 *A. m. iberiensis* and 15 C‐lineage individuals chosen at random (25% of the total sample size), for subsequent assay validation (Table 1). The remaining 88 *A. m. iberiensis* and 44 C‐lineage individuals (23 *A. m. carnica* and 21 *A. m. ligustica*) were used as the training data set for selecting informative SNPs.

**Table 1:** Sample sizes of training and holdout data sets for each population

Population	Training set	Holdout set	Total
*Apis mellifera iberiensis*	88	29	117
C‐lineage *(A. m. carnica* & *A. m. ligustica)*	44 (23 + 21)	15 (5 + 10)	59 (28 + 31)
Total	132	44	176

The most informative SNPs were identified from *F*_ST_ values (fixed SNPs, *F*_ST_ = 1), calculated in PLINK between *A. m*. *iberiensis* and C‐lineage individuals using the training data set. To uncover the putative functional role of the highly differentiated SNPs, we used SNPeff 4.3 (Cingolani et al., 2012) and the NCBI honeybee annotation version 102 (Pruitt et al., 2013). Subsequently, we performed a gene ontology (GO) analysis in the DAVID v.8.0 database (Huang, Sherman, & Lempicki, 2009) considering the GO terms of the biological process (BP), molecular function (MF), cellular component (CC) (Gene Ontology Consortium, 2015) and the KEGG pathway (Kanehisa, Sato, Kawashima, Furumichi, & Tanabe, 2016).

To downsize the number of fixed SNPs, the first filter eliminated SNPs <5,000 bp apart, which carry redundant information (Figure 2). This distance threshold correlates with the high linkage disequilibrium (LD) decay in honeybees (Wallberg, Glémin, & Webster, 2015) and has been used by others (Chapman et al., 2015; Harpur et al., 2014). In this filtering step, SNPs located in 3′UTR, 5′UTR, missense, splice donor and splice regions were preferentially retained to assure that the reduced assays included SNPs of putative functional relevance and thereby represent real phenotypic differences between lineages.

The subsequent filtering step was linked to the Agena Bioscience MassARRAY^®^ MALDI‐TOF genotyping system (Figure 2). To increase the probability of amplification success, we removed the SNPs which had >5 variable nucleotides on either side of the 250 bp flanking sequences, which will be used for primer design (Table S1). Additionally, SNPs located in ambiguous regions of the reference genome were excluded using the following criteria: (i) >5 sequential unknown nucleotides (N) in the flanking regions, (ii) flanking regions matching multiple contigs on the reference genome and (iii) flanking regions consisted of short repeats. The remaining SNPs were used to design four multiplexes (M1, M2, M3 and M4) with the software Assay Design 4.0 (http://www.agenabio.com), which selects the best combination of SNPs for amplification by preventing hairpin and dimer formation. Three criteria were followed to construct each multiplex (hereafter termed reduced SNP assay) aiming at a maximum of 40 SNPs per multiplex, as allowed by the MassARRAY^®^ technology: (i) every chromosome represented, (ii) at least four putative functional SNPs and (iii) no overlapping SNPs between multiplexes. For comparison purposes, we also constructed four assays of randomly chosen SNPs (hereafter termed random SNP assays) from the whole‐genome data set with the same size of the four multiplexes.

### 2.4 Assay validation

For validating the reduced SNP assays, we simulated hybrid haplotypes using the software admix‐simu (https://github.com/williamslab/admix-simu) and a window‐based 100‐kbp resolution recombination map from Wallberg et al. (2015). To avoid related haplotypes in the simulated F1 and backcross haplotypes, we used the parental individuals only once in the simulation of recombination. The 29 *A. m. iberiensis* and the 15 C‐lineage individuals of the holdout data set were randomly chosen to simulate the hybrid haplotypes as follows: F1s were simulated using 15 *A. m. iberiensis* and 15 C‐lineage individuals as parents; backcrosses were simulated using 14 F1 and the remaining 14 *A. m. iberiensis* individuals as parents.

The reduced and random SNP assays were validated in the holdout (*N* = 44) and simulated data sets (*N* = 29) by estimating the Q‐values with ADMIXTURE, using the unsupervised option and the default settings, for *K* = 2 and 200 bootstrap replicates. We examined the performance of each reduced and random SNP assay (individually or by combining the best performing assays) against the whole‐genome data set, which provides the true Q‐value, by calculating (i) deviation, (ii) precision and (iii) accuracy. Precision was assessed by the Pearson correlation coefficient (*r*) and the standard deviation of the differences. Accuracy was assessed through the percentage of absolute error.

## 3 RESULTS

### 3.1 SNP calling and population structure

A total of 2,366,382 SNPs were detected in the whole‐genome sequences of 176 individuals (117 *A. m. iberiensis*, 31 *A. m. ligustica* and 28 *A. m. carnica*), with a genotyping rate of 0.986. Information on sample origin, coverage and variant calling statistics is provided in Table S2. Using the whole‐genome sequences, the global pairwise F_ST_ values were estimated for the M‐lineage *A. m. iberiensis* and the C‐lineage *A. m. carnica* and *A. m. ligustica* (Table 2). As expected, *F*_ST_ between the subspecies belonging to the highly divergent M and C lineages was high (*F*_ST_ ≥0.53), whereas between the closely related *A. m. carnica* and *A. m. ligustica* was low (*F*_ST_ = 0.06). The two lineages are clearly separated at the optimal *K* = 2 (Figure S1), with the 117 *A. m. iberiensis* individuals forming one cluster and the 28 *A. m. carnica* together with the 31 *A. m. ligustica* individuals forming another cluster (Figure S2).

**Table 2:** Population differentiation estimated from average genomewide F_ST_

Population	*Apis mellifera carnica*	*A. m. ligustica*	C‐lineage (*A. m. carnica* & *A. m. ligustica*)
*A. m. iberiensis*	0.540	0.549	0.532
*A. m. ligustica*	0.061		

### 3.2 Effect of sampling bias on the number of fixed SNPs

The effect of sample size and sampling a geographically restricted area on the number of fixed SNPs (*F*_ST_ = 1) was examined to understand to what extent false‐positive fixed SNPs would bias reduced SNP assays for estimating introgression. A total of 11,091 fixed SNPs were detected between the complete *A. m. iberiensis* data set (*N* = 117) and the C‐lineage data set (*N* = 59). As expected, the number of fixed SNPs and the number of false positives increases as the *A. m. iberiensis* sample size decreases, and this trend is more pronounced when *N* < 25 (Table 3). For *N* = 5, a large proportion of false positives (33.9%) displayed a *F*_ST_ ≤0.95 with a minimum of 0.084, which might impact the power of reduced SNP assays. However, the impact is negligible for *N* ≥ 25 as the proportion of false positives is ≤3.4% and the minimum *F*_ST_ value (0.695) is still relatively high (Table 3).

**Table 3:** Fixed SNPs and 95% confidence interval (CI) estimated from random subsets of variable sample size (five replicates each) of *Apis mellifera iberiensis* and statistics for *F*_ST_ values estimated from the false‐positive fixed SNPs

Sample size subset	Mean number of fixed SNPs (±95% CI)	Mean number of false‐positive fixed SNPs^a^	Mean % of false‐positive fixed SNPs with an *F*_ST_ ≤0.95^b^	Mean minimum *F*_ST_
5	25,428 (±1,184)	14,337	33.9	0.084
10	18,878 (±354)	7,787	14	0.334
25	15,700 (±127)	4,609	3.4	0.695
50	13,784 (±282)	2,693	0.3	0.880
75	12,480 (±306)	1,389	0.1	0.942
100	11,736 (±165)	645	0	0.970

a Calculated by subtracting the number of fixed SNPs estimated for each sample size subset from 11,091 fixed SNPs estimated for the complete data set of *A. m. iberiensis* (*N* = 117), which displays a minimum *F*_ST_ = 1.

b Calculated by retrieving the *F*_ST_ values obtained from the complete *A. m. iberiensis* data set for the false positives and calculating the percentage with a *F*_ST_ ≤0.95.

Sampling a geographically restricted area also influences the number of fixed SNPs, although the extent of bias depends on sample origin (Table 4). Interestingly, the highest number of false positives is identified when sampling is restricted to Portugal (PT). In contrast, sampling along the north–south transect in the centre of Iberia (CT) provides the best estimate of fixed SNPs. Considering the percentage of false positives with a *F*_ST_ ≤0.95, the best result was obtained for the IP subset with only 10.4% and with a minimum value of *F*_ST_ = 0.763. This contrasted with the PT subset for which there were twice as many (20.2%) false positives with a *F*_ST_ ≤0.95 and a considerably lower minimum value of 0.275 (Table 4).

**Table 4:** Fixed SNPs estimated from geographical subsets of *Apis mellifera iberiensis* and statistics for *F*_ST_ values estimated from the false‐positive fixed SNPs

Geographical subset^a^	Number of fixed SNPs	Number of false‐positive fixed SNPs^b^	% of false‐positive fixed SNPs with an *F*_ST_ ≤0.95^c^	Minimum *F*_ST_
PT	17,738	6,647	20.2	0.275
CT	15,009	3,918	13.7	0.700
MT	15,384	4,293	11.8	0.676
IP	15,371	4,280	10.4	0.763

a PT, Portugal; CT, Central transect; MT, Mediterranean transect; IP, Iberian Peninsula.

b Calculated by subtracting the number of fixed SNPs estimated for each geographical subset from 11,091 fixed SNPs estimated for the complete data set of *A. m. iberiensis* (*N* = 117), which displays a minimum *F*_ST_ = 1.

c Calculated by retrieving the *F*_ST_ values obtained from the complete *A. m. iberiensis* data set for the false positives and calculating the percentage with a *F*_ST_ ≤0.95.

### 3.3 Selection and genomic information of highly informative SNPs

Having assessed the potential effects of sampling bias, we were able to follow Anderson's simple training and holdout method without incorporating a significant bias when selecting highly informative SNPs (Figure 2). Accordingly, highly informative SNPs for estimating C‐lineage introgression into *A. m. iberiensis* were selected using the training data set (88 *A. m. iberiensis* and 44 C‐lineage individuals). A total of 18,272 SNPs were fixed (*F*_ST_ = 1) (Table S3, Figure S3), an increase of 7,181 fixed SNPs compared to that calculated from the complete data set (117 *A. m. iberiensis* data set and 59 C‐lineage individuals). While these SNPs were not fixed in the complete data set, they were still highly differentiated (*F*_ST_ ≥0.95 for 98.9% of the SNPs; minimum *F*_ST_ = 0.925) and thereby highly informative.

The 18,272 SNPs were distributed across the 16 honeybee chromosomes (Figure S3) and located in 247 intergenic regions and 1,347 genic regions (±5 kb around coding sequences; Table S3). Chromosome 11 contained the highest proportion of fixed SNPs (3.1%, 4,729 SNPs), whereas chromosome 7 had the least (0.3%, 400 SNPs; Table S4). The physical distance between the fixed SNPs ranged from 1 to 2,587,074 bp with a mean of 11,261 bp. Most fixed SNPs are located in introns (7,666) and intergenic regions (4,257); however, a number are located in regions of putative functional relevance, including 47 SNPs (distributed along 37 genes) that are nonsynonymous or missense variants (Table S5). Of the 1,347 genic regions containing SNPs, 12 harbour more than 100 SNPs (Table S6). Gene ontology (GO) analysis revealed 13 significantly enriched functional terms (modified Fisher exact *p*‐value <.05; Table S7). The biological processes term “regulation of transcription, DNA‐templated” shared 12 genes with the molecular function term, “transcription factor activity, sequence‐specific DNA binding.” Two other molecular function terms are associated with more than 26 genes related to DNA binding (“sequence‐specific DNA binding,” “DNA binding”). The KEGG pathways were represented by four terms “aminoacyl‐tRNA biosynthesis,” “Wnt signalling pathway,” “mRNA surveillance pathway” and “insulin resistance.”

### 3.4 Assay design

**Figure 3:**
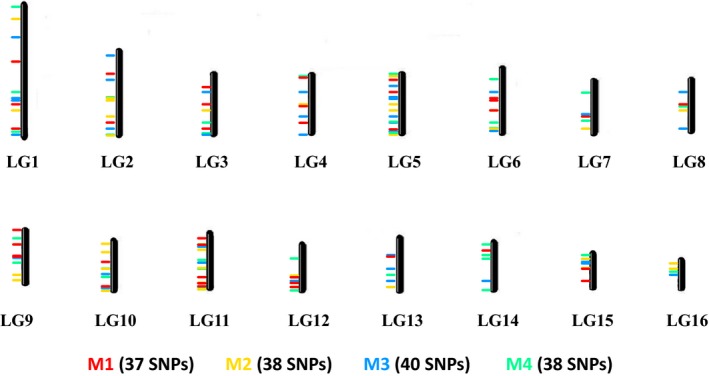
Chromosome map showing the SNP positions of the four reduced assays (M1–M4)

Several filters were applied to the initial 18,272 fixed SNPs identified in the training data set, resulting in a final data set of 708 SNPs, which were used to design four multiplexes (or reduced assays) with the assay design tool of Agena (Figure 2). The resulting assays contained 37 (M1), 38 (M2), 40 (M3) and 38 (M4) SNPs (Table S8). Each assay combines highly informative SNPs covering 15 (M1 lacks SNPs in chromosome 16, M2 in chromosome 14) or 16 (M3, M4) chromosomes (Figure 3, Table S4).

### 3.5 Assay validation

**Figure 4:**
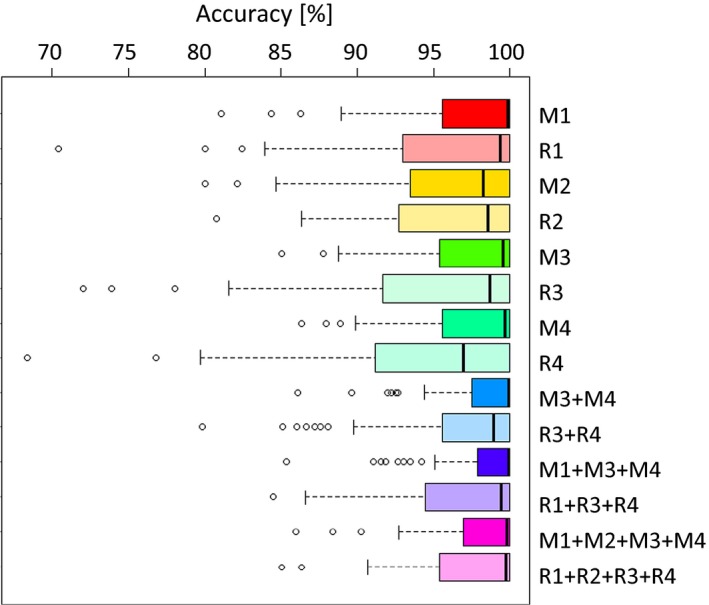
Accuracy of single and combined reduced (M1–M4) and random (R1–R4) SNP assays. The box denotes the first and third quartiles and median accuracy marked with a bold vertical line within the box. Outliers are indicated by circles. Random assays consistently have a larger interquartile range than the corresponding reduced assay

The reduced (M1, M2, M3, M4) and random SNP assays (R1, R2, R3, R4) were validated in the holdout (29 *A. m. iberiensis*) and simulated (29 hybrid haplotypes) data sets (Figure 2). The Q‐values estimated using the eight SNP assays, or their combinations, were compared with those obtained from the whole‐genome data set (2.336 M SNPs), which is assumed to provide the true admixture proportions. The Q‐values obtained with M1, M2, M3 and M4 are highly correlated with those of the whole‐genome data set (.956 < *r* < .982; Table 5, Figure S4). While all statistics indicate that the four reduced assays have a good performance, M2 shows consistently the worst behaviour. The mean accuracy, for example, is high across the assays, varying between 95.93% (M2) and 97.42% (M1), but the dispersion is much greater for M2 (Table 5, Figure 4).

**Table 5:** Performance of the reduced (M1–M4) and random (R1–R4) SNP assays in estimating C‐lineage introgression (Q‐values) of holdout and simulated data sets as compared to the whole‐genome data set

Assay	# of SNPs	Pearson's *r* (95% CI)	Standard error	Mean error	# Ind error >0.05	Max error	% Mean accuracy	Precision	Pure classified as hybrid	Hybrid classified as pure
		(i)	(ii)	(iii)	(iv)	(v)	(vi)	(vii)	(viii)	
M1	37	0.975 (0.958–0.985)	0.046	0.026	12	0.189	97.42	0.043	0	0
R1	37	0.949 (0.915–0.970)	0.069	0.043	20	0.296	95.71	0.062	1	3
M2	38	0.956 (0.927–0.974)	0.046	0.041	20	0.200	95.93	0.053	1	0
R2	38	0.967 (0.945–0.981)	0.075	0.037	20	0.192	96.34	0.047	3	1
M3	40	0.978 (0.964–0.987)	0.048	0.028	13	0.150	97.24	0.038	0	0
R3	40	0.933 (0.888–0.960)	0.067	0.05	14	0.279	95.04	0.069	1	1
M4	38	0.982 (0.969–0.989)	0.044	0.026	13	0.137	97.41	0.036	1	0
R4	38	0.925 (0.876–0.955)	0.062	0.053	22	0.316	94.71	0.069	3	1
M3 + M4	78	0.988 (0.979–0.993)	0.04	0.018	9	0.139	98.18	0.030	0	0
R3 + R4	78	0.967 (0.945–0.981)	0.051	0.034	13	0.201	96.62	0.049	1	0
M1 + M3 + M4	115	0.987 (0.979–0.993)	0.037	0.018	8	0.147	98.15	0.030	0	0
R1 + R3 + R4	115	0.976 (0.959–0.986)	0.046	0.03	16	0.155	97.01	0.041	0	1
M1 + M2 + M3 + M4	153	0.986 (0.977–0.992)	0.003	0.02	9	0.140	98.02	0.031	0	0
R1 + R2 + R3 + R4	153	0.981 (0.967–0.989)	0.042	0.027	14	0.150	97.35	0.037	0	1

(i) Pearson's correlation coefficient *r*; (ii) mean standard error estimated from 200 bootstrap replicates by ADMIXTURE; (iii) mean error calculated by the absolute difference; (iv) number of individuals with error >0.05; (v) maximum error; (vi) mean accuracy calculated via percentage of absolute error; (vii) precision defined as the standard deviation of the absolute error; (viii) number of misclassified individuals (Q‐value threshold of 0.05).

Interestingly, the four random SNP assays also show a good performance, although M3 and M4 are considerably better, as indicated by the nonoverlapping confidence intervals of the correlations (Table 5, Figure S4) and the lower dispersion of the accuracy values around the median (Figure 4). Another important difference between M and R assays arises from the misclassification of individuals and simulated haplotypes (pure classified as hybrid and vice versa), with the reduced assays performing consistently better than the random ones. For example, all random assays misclassified between one to three pure individuals as hybrids, which never occurred with the reduced assays (Tables 5, S9).

The overall performance increases when the reduced assays are combined (Tables 5, S9; Figures 4, S4). The best result is obtained for the combination of M1, M3 and M4, which represents a total of 115 highly informative SNPs distributed across the 16 chromosomes. However, the combination of M3 and M4, with only 78 SNPs, was nearly as good (Table 5). In summary, while there is an increment in the overall performance when combining M1, M3 and M4, their individual use still provides robust estimates of C‐lineage introgression into *A. m. iberiensis*.

## 4 DISCUSSION

Developing cost‐effective molecular tools for accurate estimation of introgression in *A. mellifera* is increasingly important as commercial strains (mostly of C‐lineage ancestry) are threatening native genetic diversity in many regions throughout Europe (Bertrand et al., 2015; De la Rúa et al., 2009; Jensen et al., 2005; Parejo et al., 2016; Pinto et al., 2014; Soland‐Reckeweg et al., 2009). In the postgenomics era, rapid innovations in high‐throughput sequencing technologies make it possible to construct extensive whole‐genome data sets, especially in model organisms with small genomes like the honeybee (Weinstock et al., 2006). However, while whole‐genome sequencing is increasingly inexpensive (~200 €/honeybee), it is still not affordable for conservation management applications. Furthermore, the processing of the large amounts of data generated by whole‐genome sequencing requires bioinformatics expertise and powerful computational resources typically not available to state entities or conservation centres. Whole‐genome sequences, however, can be used to generate baseline data for developing robust molecular tools for routine genotyping hundreds of samples in a time‐ and cost‐effective manner. Here, we mined a massive whole‐genome data set, representing the focal *A. m. iberiensis* and the two C‐lineage subspecies (*A. m. carnica* and *A. m. ligustica*) preferred worldwide in commercial breeding, to identify fixed SNPs for constructing robust reduced assays. While *A. m. iberiensis* and *A. m. ligustica* were sampled across their entire native range, most of *A. m. carnica* samples were from areas in Switzerland where beekeepers have kept this subspecies. Nevertheless, these samples are good representatives of *A. m. carnica*, as revealed by admixture proportions greater than 0.95 inferred from whole genomes. By employing very stringent sample selection and SNP filtering criteria, our approach represents a rigorous methodological example that can be applied for developing reduced SNP assays in any other organism.

### 4.1 Effect of sampling bias on the number of fixed SNPs

Considering the long‐standing problem of ascertainment bias during discovery and selection of informative SNPs (Albrechtsen, Nielsen, & Nielsen, 2010, and references therein), we started by testing the effect of sample size and sampling breadth on the number of SNPs erroneously identified as fixed between *A. m. iberiensis* and C‐lineage (false‐positive fixed SNPs). We found that limited sample size can be problematic, as a considerable number of false‐positive fixed SNPs with *F*_ST_ ≤0.95 could negatively impact the development of a sensitive SNP assay. This effect is reduced for *N* = 25, and increasing sample size above 50 yields diminishing returns in fixed SNPs, suggesting that an optimal cost–benefit ratio is reached. Beyond this point, further increasing sample size will likely lead to detection of new SNPs in the population. However, such low‐frequency SNPs (i.e., singletons) are not of concern for discriminating populations nor for identifying highly informative SNPs.

A bias is also introduced when sampling a geographically restricted area. From the three geographic subsets examined, the Portuguese revealed the highest number of false positives while the Central and Mediterranean behaved similarly to the subset covering the entire Iberian honeybee range. While both the Central and Mediterranean subsets cover the north‐eastern–south‐western Iberian cline, the Portuguese subset represents a small portion of the *A. m. iberiensis* genetic complexity (Chávez‐Galarza et al., 2015, 2017; Pinto et al., 2013). But more importantly, this subset generated a substantial number of false positives with a lower differentiation power (Table 4). As a consequence, reduced SNP assays designed from samples strictly originating from Portugal would not be appropriate to discriminate *A. m. iberiensis* from C‐lineage, but only the Portuguese populations. While selecting informative SNPs from geographically limited samples or subpopulations may be valid for very specific applications, it is not a recommended procedure in most cases (especially when knowledge on population structure is lacking) and questions the wider applicability of SNP assays. It is well established that this kind of ascertainment bias influences population genetic measures such as divergence (Albrechtsen et al., 2010) and demography (Morin, Luikart, Wayne, & Grp, 2004; Wakeley, Nielsen, Liu‐Cordero, & Ardlie, 2001). Accordingly, we assured a sufficiently large and representative sample of the *A. m. iberiensis* diversity, which covers the Iberian cline, for developing accurate reduced assays while at the same time leaving independent holdout samples for validation.

### 4.2 Genomic information of the highly informative SNPs

A large number of SNPs (18,272) were fixed between *A. m. iberiensis* and C‐lineage subspecies. This was an expected result because M and C are the most divergent of the four lineages (Wallberg et al., 2014). The top enriched GO terms of the genes marked by those SNPs were associated with numerous genes related to regulation of expression, which is essential for the versatility and adaptability of a species for short‐ and long‐term environmental changes (López‐Maury, Marguerat, & Bahler, 2008). This is consistent with the complex evolutionary history of *A. mellifera* and its numerous subspecies, which have adapted to the diversity of habitats and climates in its large distributional range (Harpur et al., 2014; Wallberg et al., 2014).

### 4.3 Assay design and validation

Having a large number of fixed SNPs is an enormous advantage when designing reduced SNP assays, as they represent ideal ancestry informative markers (Rosenberg, Li, Ward, & Pritchard, 2003). Yet, the overall high differentiation between *A. m. iberiensis* and C‐lineage honeybees explains why all tested assays, including those constructed from randomly selected SNPs, performed well. For example, a random set of 153 SNPs performed equally well as the 153 fixed SNPs across the four reduced assays. This was also shown by Pardo‐Seco, Martinón‐Torres, and Salas (2014) who concluded that it is not primarily individual informativeness, but the number of markers that plays a major role in accurately estimating genome ancestry. Although all the assays show a remarkable performance on average, we highlight, however, that differences arise at the individual level. While average statistics can be useful for measuring the admixture proportions of an entire population, they are not adequate to support decision‐making at the individual level, for example when choosing individuals for conservation breeding purposes. Three random assays had individual errors >25% compared to the whole‐genome information, which is far from acceptable in a context of conservation. Moreover, pure *A. m. iberiensis*, which were misclassified as hybrids, could lead to exclusion of individuals with valuable and unique genetic components.

Apart from assay performance, the genotyping cost is another important criterion to take into consideration. Genotyping with the MassARRAY^®^ system costs approximately 5.5€ per individual and single assay. While the M1, M3 and M4 perform remarkably well, the minimal individual error and the highest accuracy are achieved when combining the three assays (115 SNPs), although the combination of M3 and M4 (78 SNPs) is nearly as good. The choice of using up to three assays is ultimately dictated by budget constraints; nevertheless, an interesting trade‐off between accuracy and cost is achieved when genotyping the 78 SNPs.

Unlike many populations of *A. m. mellifera* from western Europe and *A. m. iberiensis* from the archipelagos of Baleares and Macaronesia*,* which are threatened by human‐mediated gene flow (De la Rúa et al., 2001, 2003; Jensen et al., 2005; Miguel et al., 2015; Muñoz et al., 2014; Pinto et al., 2014), there is very limited introgression in *A. m. iberiensis* populations of Iberia (Chávez‐Galarza et al., 2015). Therefore, it is crucial to monitor Iberian populations, before gene complexes shaped by natural selection over evolutionary time are irretrievably lost. Here, we took advantage of whole‐genome sequence data, which provided millions of SNPs, to design highly powerful assays containing a low number of SNPs capable of estimating C‐lineage introgression into *A. m. iberiensis* with a high level of accuracy. We recommend the combination of the best two (78 SNPs) or three (115 SNPs) reduced SNP assays, although one assay can also be used when there are budget constraints. These assays can be used to estimate C‐lineage introgression not only in the native range of *A. m. iberiensis* in Iberia but also in the introduced range in the archipelagos of Baleares and Macaronesia, and in South America.

This study provides a powerful set of tools to safeguard a unique legacy of honeybee diversity for future generations. While these tools can only be applied to honeybees, the approach demonstrated herein (from testing the effect of sampling bias to the intricate steps involved in the design of the reduced SNP assays) is of high general value in a wide range of scenarios for the conservation of potentially hybridized domestic and wildlife populations.

## DATA ACCESSIBILITY

SNPs for the 176 individuals in vcf format are available from the Dryad Digital Repository: https://doi.org/10.5061/dryad.v8cp134.

## References

[eva12623-bib-0001] Albrechtsen, A. , Nielsen, F. C. , & Nielsen, R. (2010). Ascertainment biases in SNP chips affect measures of population divergence. Molecular Biology and Evolution, 27(11), 2534–2547. 10.1093/molbev/msq148 20558595 PMC3107607

[eva12623-bib-0002] Alexander, D. H. , Novembre, J. , & Lange, K. (2009). Fast model‐based estimation of ancestry in unrelated individuals. Genome Resources, 19, 1655–1664. 10.1101/gr.094052.109 PMC2752134 19648217

[eva12623-bib-0003] Amirisetty, S. , Khurana Hershey, G. K. , & Baye, T. M. (2012). AncestrySNPminer: A bioinformatics tool to retrieve and develop ancestry informative SNP panels. Genomics, 100(1), 57–63. 10.1016/j.ygeno.2012.05.003 22584067 PMC3433799

[eva12623-bib-0004] Anderson, E. C. (2010). Assessing the power of informative subsets of loci for population assignment: Standard methods are upwardly biased. Molecular Ecology Resources, 10(4), 701–710. 10.1111/j.1755-0998.2010.02846.x 21565075

[eva12623-bib-0005] Arias, M. C. , Rinderer, T. E. , & Sheppard, W. S. (2006). Further characterization of honey bees from the Iberian Peninsula by allozyme, morphometric and mtDNA haplotype analyses. Journal of Apicultural Research, 45(4), 188–196. 10.1080/00218839.2006.11101346

[eva12623-bib-0006] Bertrand, B. , Alburaki, M. , Legout, H. , Moulin, S. , Mougel, F. , & Garnery, L. (2015). MtDNA COI‐COII marker and drone congregation area: An efficient method to establish and monitor honeybee (*Apis mellifera* L.) conservation centres. Molecular Ecology Resources, 15, 673–683. 10.1111/1755-0998.12339 25335970

[eva12623-bib-0007] Büchler, R. , Costa, C. , Hatjina, F. , Andonov, S. , Meixner, M. D. , Le Conte, Y. , … Wilde, J. (2014). The influence of genetic origin and its interaction with environmental effects on the survival of *Apis mellifera* L. Colonies in Europe. Journal of Apicultural Research, 53, 205–214. 10.3896/IBRA.1.53.2.03

[eva12623-bib-0008] Chang, C. C. , Chow, C. C. , Tellier, L. C. , Vattikuti, S. , Purcell, S. M. , & Lee, J. J. (2015). Second‐generation PLINK: Rising to the challenge of larger and richer datasets. Gigascience, 4, 7 10.1186/s13742-015-0047-8 25722852 PMC4342193

[eva12623-bib-0009] Chapman, N. C. , Harpur, B. A. , Lim, J. , Rinderer, T. E. , Allsopp, M. H. , Zayed, A. , & Oldroyd, B. P. (2015). A SNP test to identify Africanized honeybees via proportion of ‘African’ ancestry. Molecular Ecology Resources, 15(6), 1346–1355. 10.1111/1755-0998.12411 25846634

[eva12623-bib-0010] Chávez‐Galarza, J. , Garnery, L. , Henriques, D. , Neves, C. J. , Loucif‐Ayad, W. , Jonhston, J. S. , & Pinto, M. A. (2017). Mitochondrial DNA variation of *Apis mellifera iberiensis*: Further insights from a large‐scale study using sequence data of the tRNAleu‐cox2 intergenic region. Apidologie, 48, 533–544. 10.1007/s13592-017-0498-2

[eva12623-bib-0011] Chávez‐Galarza, J. , Henriques, D. , Johnston, J. S. , Azevedo, J. C. , Patton, J. C. , Muñoz, I. , … Pinto, M. A. (2013). Signatures of selection in the Iberian honey bee (*Apis mellifera iberiensis*) revealed by a genome scan analysis of single nucleotide polymorphisms. Molecular Ecology, 22(23), 5890–5907. 10.1111/mec.12537 24118235

[eva12623-bib-0012] Chávez‐Galarza, J. , Henriques, D. , Johnston, J. S. , Carneiro, M. , Rufino, J. , Patton, J. C. , & Pinto, M. A. (2015). Revisiting the Iberian honey bee (*Apis mellifera iberiensis*) contact zone: Maternal and genome‐wide nuclear variations provide support for secondary contact from historical refugia. Molecular Ecology, 24(12), 2973–2992. 10.1111/mec.13223 25930679

[eva12623-bib-0013] Chen, C. , Liu, Z. , Pan, Q. , Chen, X. , Wang, H. , Guo, H. , … Shi, W. (2016). Genomic analyses reveal demographic history and temperate adaptation of the newly discovered honey bee subspecies *Apis mellifera sinisxinyuan* n. ssp. Molecular Biology and Evolution, 33(5), 1337–1348. 10.1093/molbev/msw017 26823447 PMC4839221

[eva12623-bib-0014] Cingolani, P. , Platts, A. , Wang, L. L. , Coon, M. , Nguyen, T. , Wang, L. , … Ruden, D. M. (2012). A program for annotating and predicting the effects of single nucleotide polymorphisms, SnpEff: SNPs in the genome of *Drosophila melanogaster* strain w1118; iso‐2; iso‐3. Fly, 6(2), 80–92. 10.4161/fly.19695 22728672 PMC3679285

[eva12623-bib-0015] De la Rúa, P. , Galián, J. , Serrano, J. , & Moritz, R. (2001). Genetic structure and distinctness of *Apis mellifera* L. populations from the Canary Islands. Molecular Ecology, 10(7), 1733–1742. 11472540 10.1046/j.1365-294x.2001.01303.x

[eva12623-bib-0016] De la Rúa, P. , Galián, J. , Serrano, J. , & Moritz, R. F. (2003). Genetic structure of Balearic honeybee populations based on microsatellite polymorphism. Genetics Selection Evolution, 35(3), 339 10.1051/gse:2003012 PMC2732703 12729553

[eva12623-bib-0017] De la Rúa, P. , Jaffé, R. , Dall'Olio, R. , Muñoz, I. , & Serrano, J. (2009). Biodiversity, conservation and current threats to European honeybees. Apidologie, 40, 263–284. 10.1051/apido/2009027

[eva12623-bib-0018] De la Rúa, P. , Jaffé, R. , Muñoz, I. , Serrano, J. , Moritz, R. F. , & Kraus, F. B. (2013). Conserving genetic diversity in the honeybee: Comments on Harpur et al. (2012). Molecular Ecology, 22(12), 3208–3210. 10.1111/mec.12333 24433572

[eva12623-bib-0019] Ding, L. , Wiener, H. , Abebe, T. , Altaye, M. , Go, R. C. , Kercsmar, C. , … Baye, T. M. (2011). Comparison of measures of marker informativeness for ancestry and admixture mapping. BMC Genomics, 12(1), 622 10.1186/1471-2164-12-622 22185208 PMC3276602

[eva12623-bib-0020] Engel, M. S. (1999). The taxonomy of recent and fossil honey bees (Hymenoptera: Apidae; Apis). Jounal of Hymenoptera Research, 8(2).

[eva12623-bib-0021] van Engelsdorp, D. , & Meixner, M. D. (2010). A historical review of managed honey bee populations in Europe and the United States and the factors that may affect them. Journal of Invertebrate Pathology, 103(Suppl.), S80–S95. 10.1016/j.jip.2009.06.011 19909973

[eva12623-bib-0022] Francis, R. M. , Amiri, E. , Meixner, M. D. , Kryger, P. , Gajda, A. , Andonov, S. , … Wilde, J. (2014). Effect of genotype and environment on parasite and pathogen levels in one apiary – A case study. Journal of Apicultural Research, 53(2), 230–232. 10.3896/IBRA.1.53.2.14

[eva12623-bib-0023] Franck, P. , Garnery, L. , Solignac, M. , & Cornuet, J.‐M. (1998). The origin of West European subspecies of honeybees (*Apis mellifera*): New insights from microsatellite and mitochondrial data. Evolution, 52(4), 1119–1134. 10.2307/2411242 28565209

[eva12623-bib-0024] Frankham, R. , Ballou, J. D. , & Briscoe, D. A. (2002). Introduction to conservation genetics. Cambridge, UK: Cambridge University Press.

[eva12623-bib-0025] Gene Ontology Consortium (2015). Gene ontology consortium: Going forward. Nucleic Acids Research, 43(D1), D1049–D1056. 10.1093/nar/gku1179 25428369 PMC4383973

[eva12623-bib-0026] Harpur, B. A. , Kent, C. F. , Molodtsova, D. , Lebon, J. M. , Alqarni, A. S. , Owayss, A. A. , & Zayed, A. (2014). Population genomics of the honey bee reveals strong signatures of positive selection on worker traits. Proceedings of the National Academy of Sciences of the United States of America, 111(7), 2614–2619. 10.1073/pnas.1315506111 24488971 PMC3932857

[eva12623-bib-0027] Huang, D. W. , Sherman, B. T. , & Lempicki, R. A. (2009). Bioinformatics enrichment tools: Paths toward the comprehensive functional analysis of large gene lists. Nucleic Acids Research, 37(1), 1–13. 10.1093/nar/gkn923 19033363 PMC2615629

[eva12623-bib-0028] Hulsegge, B. , Calus, M. , Windig, J. , Hoving‐Bolink, A. , Maurice‐van Eijndhoven, M. , & Hiemstra, S. (2013). Selection of SNP from 50K and 777K arrays to predict breed of origin in cattle. Journal of Animal Science, 91(11), 5128–5134. 10.2527/jas.2013-6678 24045484

[eva12623-bib-0029] Jensen, A. B. , Palmer, K. A. , Boomsma, J. J. , & Pedersen, B. V. (2005). Varying degrees of *Apis mellifera ligustica* introgression in protected populations of the black honeybee, *Apis mellifera mellifera*, in northwest Europe. Molecular Ecology, 14(1), 93–106. 10.1111/j.1365-294X.2004.02399.x 15643954

[eva12623-bib-0030] Judge, M. , Kelleher, M. , Kearney, J. , Sleator, R. , & Berry, D. (2017). Reduced genotype panels for breed assignment of Angus and Hereford cattle. Animal, 11(6), 938–947. 10.1017/S1751731116002457 27881206

[eva12623-bib-0031] Kanehisa, M. , Sato, Y. , Kawashima, M. , Furumichi, M. , & Tanabe, M. (2016). KEGG as a reference resource for gene and protein annotation. Nucleic Acids Research, 44(Database issue), D457–D462. 10.1093/nar/gkv1070 26476454 PMC4702792

[eva12623-bib-0032] Karlsson, S. , Moen, T. , Lien, S. , Glover, K. A. , & Hindar, K. (2011). Generic genetic differences between farmed and wild Atlantic salmon identified from a 7K SNP‐chip. Molecular Ecology Resources, 11(s1), 247–253. 10.1111/j.1755-0998.2010.02959.x 21429178

[eva12623-bib-0033] Le Conte, Y. , & Navajas, M. (2008). Climate change: Impact on honey bee populations and diseases. Revue Scientifique et Technique‐Office International des Epizooties, 27, 499–510. 18819674

[eva12623-bib-0034] López‐Maury, L. , Marguerat, S. , & Bahler, J. (2008). Tuning gene expression to changing environments: From rapid responses to evolutionary adaptation. Nature Reviews Genetics, 9, 583–593. 10.1038/nrg2398 18591982

[eva12623-bib-0035] Mariette, S. , Le Corre, V. , Austerlitz, F. , & Kremer, A. (2002). Sampling within the genome for measuring within‐population diversity: Trade‐offs between markers. Molecular Ecology, 11(7), 1145–1156. 10.1046/j.1365-294X.2002.01519.x 12074722

[eva12623-bib-0036] Meixner, M. D. , Costa, C. , Kryger, P. , Hatjina, F. , Bouga, M. , Ivanova, E. , & Büchler, R. (2010). Conserving diversity and vitality for honey bee breeding. Journal of Apicultural Research, 49(1), 85–92.

[eva12623-bib-0037] Meixner, M. D. , Leta, M. A. , Koeniger, N. , & Fuchs, S. (2011). The honey bees of Ethiopia represent a new subspecies of *Apis mellifera‐ Apis mellifera simensis* n. ssp. Apidologie, 42, 425–437. 10.1007/s13592-011-0007-y

[eva12623-bib-0038] Miguel, I. , Garnery, L. , Iriondo, M. , Baylac, M. , Manzano, C. , Steve Sheppard, W. , & Estonba, A. (2015). Origin, evolution and conservation of the honey bees from La Palma Island (Canary Islands): Molecular and morphological data. Journal of Apicultural Research, 54(5), 427–440.

[eva12623-bib-0039] Miguel, I. , Iriondo, M. , Garnery, L. , Sheppard, W. S. , & Estonba, A. (2007). Gene flow within the M evolutionary lineage of *Apis mellifera*: Role of the Pyrenees, isolation by distance and post‐glacial re‐colonization routes in the western Europe. Apidologie, 38(2), 141–155. 10.1051/apido:2007007

[eva12623-bib-0040] Morin, P. A. , Luikart, G. , Wayne, R. K. , & Grp, S. N. P. W. (2004). SNPs in ecology, evolution and conservation. Trends in Ecology & Evolution, 19, 208–216. 10.1016/j.tree.2004.01.009

[eva12623-bib-0041] Muñoz, I. , Henriques, D. , Jara, L. , Johnston, J. S. , Chávez‐Galarza, J. , De La Rúa, P. , & Pinto, M. A. (2017). SNPs selected by information content outperform randomly selected microsatellite loci for delineating genetic identification and introgression in the endangered dark European honeybee (*Apis mellifera mellifera*). Molecular Ecology and Resources, 17(4), 783–795. 10.1111/1755-0998.12637 27863055

[eva12623-bib-0042] Muñoz, I. , Henriques, D. , Johnston, J. S. , Chávez‐Galarza, J. , Kryger, P. , & Pinto, M. A. (2015). Reduced SNP Panels for genetic identification and introgression analysis in the dark honey bee (*Apis mellifera mellifera*). PLoS ONE, 10(4), e0124365 10.1371/journal.pone.0124365 25875986 PMC4395157

[eva12623-bib-0043] Muñoz, I. , Pinto, M. A. , & De la Rúa, P. (2014). Effects of queen importation on the genetic diversity of Macaronesian island honey bee populations (*Apis mellifera* Linneaus 1758). Journal of Apicultural Research, 53(2), 296–302.

[eva12623-bib-0044] Neumann, P. , & Blacquière, T. (2017). The Darwin cure for apiculture? Natural selection and managed honeybee health. Evolutionary Applications, 10, 226–230. 10.1111/eva.12448 28250807 PMC5322407

[eva12623-bib-0045] Pardo‐Seco, J. , Martinón‐Torres, F. , & Salas, A. (2014). Evaluating the accuracy of AIM panels at quantifying genome ancestry. BMC Genomics, 15, 543 10.1186/1471-2164-15-543 24981136 PMC4101176

[eva12623-bib-0046] Parejo, M. , Wragg, D. , Gauthier, L. , Vignal, A. , Neumann, P. , & Neuditschko, M. (2016). Using whole‐genome sequence information to foster conservation efforts for the European Dark Honey Bee, *Apis mellifera mellifera* . Frontiers in Ecology and Evolution, 4, 140 10.3389/fevo.2016.00140

[eva12623-bib-0047] Pinto, M. A. , Henriques, D. , Chávez‐Galarza, J. , Kryger, P. , Garnery, L. , van der Zee, R. , … Johnston, J. S. (2014). Genetic integrity of the Dark European honey bee (*Apis mellifera mellifera*) from protected populations: A genome‐wide assessment using SNPs and mtDNA sequence data. Journal of Apicultural Research, 53(2), 269–278. 10.3896/ibra.1.53.2.08

[eva12623-bib-0048] Pinto, M. A. , Henriques, D. , Guedes, H. , Muñoz, I. , Azevedo, J. , & De la Rúa, P. (2013). Maternal diversity patterns of Ibero‐Atlantic populations reveal further complexity of Iberian honeybees. Apidologie, 44, 430–439. 10.1007/s13592-013-0192-y

[eva12623-bib-0049] Potts, S. G. , Biesmeijer, J. C. , Kremen, C. , Neumann, P. , Schweiger, O. , & Kunin, W. E. (2010). Global pollinator declines: Trends, impacts and drivers. Trends in Ecology & Evolution, 25(6), 345–353. 10.1016/j.tree.2010.01.007 20188434

[eva12623-bib-0050] Pruitt, K. D. , Brown, G. R. , Hiatt, S. M. , Thibaud‐Nissen, F. , Astashyn, A. , Ermolaeva, O. , … McGarvey, K. M. (2013). RefSeq: An update on mammalian reference sequences. Nucleic Acids Research, 42, D756–D763. 10.1093/nar/gkt1114 24259432 PMC3965018

[eva12623-bib-0051] R Core Team (Ed.). (2016). R: A language and environment for statistical computing. Vienna, Austria: R Foundation for Statistical Computing.

[eva12623-bib-0052] Rosenberg, N. A. , Li, L. M. , Ward, R. , & Pritchard, J. K. (2003). Informativeness of genetic markers for inference of ancestry. The American Journal of Human Genetics, 73(6), 1402–1422. 10.1086/380416 14631557 PMC1180403

[eva12623-bib-0053] Ruttner, F. (1988). Biogeography and taxonomy of honey bees. Berlin, Germany: Springer.

[eva12623-bib-0054] Sheppard, W. S. , & Meixner, M. D. (2003). *Apis mellifera pomonella*, a new honey bee subspecies from Central Asia. Apidologie, 34, 367–375. 10.1051/apido:2003037

[eva12623-bib-0055] Smith, D. R. , Palopoli, M. F. , Taylor, B. R. , Garnery, L. , Cornuet, J. M. , Solignac, M. , & Brown, W. M. (1991). Geographical overlap of two mitochondrial genomes in Spanish honeybees (*Apis mellifera iberica*). Journal of Heredity, 82(2), 96–100. 2013694 10.1093/oxfordjournals.jhered.a111062

[eva12623-bib-0056] Soland‐Reckeweg, G. , Heckel, G. , Neumann, P. , Fluri, P. , & Excoffier, L. (2009). Gene flow in admixed populations and implications for the conservation of the Western honeybee, *Apis mellifera* . Journal of Insect Conservation, 13(3), 317–328. 10.1007/s10841-008-9175-0

[eva12623-bib-0057] Vignal, A. , Milan, D. , SanCristobal, M. , & Eggen, A. (2002). A review on SNP and other types of molecular markers and their use in animal genetics. Genetics Selection Evolution, 34(3), 275 10.1051/gse:2002009 PMC2705447 12081799

[eva12623-bib-0058] Wakeley, J. , Nielsen, R. , Liu‐Cordero, S. N. , & Ardlie, K. (2001). The discovery of single nucleotide polymorphisms and inferences about human demographic history. The American Journal of Human Genetics, 69, 1332–1347. 10.1086/324521 11704929 PMC1235544

[eva12623-bib-0059] Wallberg, A. , Glémin, S. , & Webster, M. T. (2015). Extreme recombination frequencies shape genome variation and evolution in the honeybee, *Apis mellifera* . PLoS Genetics, 11(4), 10.1371/journal.pgen.1005189 PMC4406589 25902173

[eva12623-bib-0060] Wallberg, A. , Han, F. , Wellhagen, G. , Dahle, B. , Kawata, M. , Haddad, N. , … Webster, M. T. (2014). A worldwide survey of genome sequence variation provides insight into the evolutionary history of the honeybee *Apis mellifera* . Nature Genetics, 46(10), 1081–1088. 10.1038/ng.3077 25151355

[eva12623-bib-0061] Weinstock, G. M. , Robinson, G. E. , Gibbs, R. A. , Worley, K. C. , Evans, J. D. , Maleszka, R. , … Wright, R. (2006). Insights into social insects from the genome of the honeybee *Apis mellifera* . Nature, 443, 931–949. 10.1038/nature05260 17073008 PMC2048586

[eva12623-bib-0062] Weir, B. S. , & Cockerham, C. C. (1984). Estimating F‐statistics for the analysis of population structure. Evolution, 38, 10.2307/2408641 28563791

[eva12623-bib-0063] Wilkinson, S. , Wiener, P. , Archibald, A. L. , Law, A. , Schnabel, R. D. , McKay, S. D. , … Ogden, R. (2011). Evaluation of approaches for identifying population informative markers from high density SNP Chips. BMC Genetics, 12(1), 45 10.1186/1471-2156-12-45 21569514 PMC3118130

