# Supplementary material for: Developing reduced SNP assays from whole‐genome sequence data to estimate introgression in an organism with complex genetic patterns, the Iberian honeybee (Apis mellifera iberiensis)
Source: Evol Appl. 2018 Mar 30;11(8):1270–82. doi: 10.1111/eva.12623 (PMC6099811; doi:10.1111/eva.12623)
Supplement: Supplementary file 1 [file EVA-11-1270-s001.docx]

**Supplementary Material and Methods**

# Developing reduced SNP assays from whole-genome sequence data to estimate introgression in an organism with complex genetic patterns, the Iberian honeybee (*Apis mellifera iberiensis*)

**SEQUENCING, Mapping, quality control, and variant calling**

Paired-end sequencing libraries (2x100/150bp) from whole-genomic DNA of single drone samples were prepared following the manufacturer’s protocol (TruSeq Nano Kit) and sequenced on an Ilumina HiSeq aiming at a depth of coverage of 10X. The choice of drones in this re-sequencing project stems from their haploidy, which enabled to obtain phased data and to confidently identify SNPs with less coverage than diploid individuals (Wragg et al., 2016). Sequencing reads were mapped against the reference genome (Amel4.5) using *bwa mem* 0.7.10 (Li & Durbin, 2009) and PCR duplicates marked using PICARD 1.80 (http://picard.sourceforge.net/). GATK 3.3.0 (McKenna et al., 2010; Van der Auwera et al., 2013) was used to improve mapping quality and read realignment around the indels. The depth of coverage (DP) statistics was calculated for each sample (bam file) with BEDtools (Quinlan & Hall, 2010).

SNP calling was performed using a two-step process following Wragg et al. (2016). Briefly, SNP variants were first identified for each sample separately applying three different variant calling tools: GATK's *UnifiedGenotyper (Van der Auwera et al., 2013)*, SAMtools’ *mpileup* 1.1 (Li et al., 2009) and PLATYPUS 0.8.1 (Rimmer et al., 2014). Variants were then filtered according to base quality (BQ) score ≥20 and mapping quality ≥30. Calls from *UnifiedGenotyper* were additionally filtered for a maximum number of two alternate alleles, genotype quality ≥30, quality by depth ≥2, and Fisher strand ≤60. After this quality control step, the three call sets were combined using BAYSIC (Cantarel et al., 2014), for the datasets of Wragg *et al.* (2017) and Parejo et al. (2016), and BCFtools (Li, 2011), for the dataset of Henriques (DH and MAP, unpublished data). Variant calling statistics was calculated for each sample with VCFtools (Danecek et al., 2011). The single-sample variant calling files (VCFs) were then merged keeping only SNP variants, which were filtered on 9≤DP≤3X mean DP to generate a set of master sites, mapped to chromosomes 1 to 16, using BCFtools. All individuals were re-genotyped with BQ≥20 at these master sites resulting in a multi-sample VCF comprising all samples.

As a quality control step, SNPs were filtered using PLINK 1.9 (Chang et al., 2015) excluding SNPs with minor allele frequency (MAF) <0.01 and genotyping call rate <0.9. Finally, functional SNP annotation was performed using SNPeff 4.1a (Cingolani et al., 2012) and the reference genome amel4.5.

**Sample selection**

A pre-requisite for developing reliable reduced SNP panels for diverse applications is to have reference samples that truly represent the original populations. For example, individuals of *A. m. carnica* showing M-lineage introgression must be removed from a reference population. With this in mind, we started with an initial whole-genome dataset of 313 individuals (Parejo et al., 2016; Wragg et al., 2017; D. Henriques, unpublished data), which included *A. m. iberiensis* (N=117), *A. m. ligustica* (N=34), *A. m. carnica* (N=37), *A. m. mellifera* (N=111), and the commercial Buckfast breed (N=14), to assess individuals’ purity using ADMIXTURE v1.3.0 (Alexander, Novembre, & Lange, 2009). The ancestry proportions of these samples were inferred using the model-based clustering for K=1 to 5 ancestral populations and the default termination criterion set to stop when the log-likelihood increases by less than 0.0001 between independent runs. At the optimal K=2, all *A. m. iberiensis* (N=117) were selected, as none of the samples was admixed, and a total of 59 C-lineage individuals (28 *A. m. carnica* and 31 *A. m. ligustica)* with <5% M-lineage ancestry were included in the final dataset. An additional filtering step of MAF <0.05 was applied on the final dataset (N=176) resulting in a whole-genome dataset of 2,366,382 SNPs.

**REFERENCES**

Alexander, D. H., Novembre, J., & Lange, K. (2009). Fast model-based estimation of ancestry in unrelated individuals. *Genome Research, 19*(9), 1655-1664. doi: 10.1101/gr.094052.109

Cantarel, B. L., Weaver, D., McNeill, N., Zhang, J., Mackey, A. J., & Reese, J. (2014). BAYSIC: A Bayesian method for combining sets of genome variants with improved specificity and sensitivity. *BMC Bioinformatics, 15*(1). doi: 10.1186/1471-2105-15-104

Chang, C. C., Chow, C. C., Tellier, L. C., Vattikuti, S., Purcell, S. M., & Lee, J. J. (2015). Second-generation PLINK: rising to the challenge of larger and richer datasets. *Gigascience, 4*, 7. doi: 10.1186/s13742-015-0047-8

Cingolani, P., Platts, A., Wang le, L., Coon, M., Nguyen, T., Wang, L., . . . Ruden, D. M. (2012). A program for annotating and predicting the effects of single nucleotide polymorphisms, SnpEff: SNPs in the genome of *Drosophila melanogaster* strain w1118; iso-2; iso-3. *Fly (Austin), 6*(2), 80-92. doi: 10.4161/fly.19695

Danecek, P., Auton, A., Abecasis, G., Albers, C. A., Banks, E., DePristo, M. A., . . . Durbin, R. (2011). The variant call format and VCFtools. *Bioinformatics, 27*(15), 2156-2158. doi: 10.1093/bioinformatics/btr330

Li, H. (2011). A statistical framework for SNP calling, mutation discovery, association mapping and population genetical parameter estimation from sequencing data. *Bioinformatics, 27*(21), 2987-2993. doi: 10.1093/bioinformatics/btr509

Li, H., & Durbin, R. (2009). Fast and accurate short read alignment with Burrows-Wheeler transform. *Bioinformatics, 25*(14), 1754-1760. doi: 10.1093/bioinformatics/btp324

Li, H., Handsaker, B., Wysoker, A., Fennell, T., Ruan, J., Homer, N., . . . Durbin, R. (2009). The Sequence Alignment/Map format and SAMtools. *Bioinformatics, 25*(16), 2078-2079. doi: 10.1093/bioinformatics/btp352

McKenna, A., Hanna, M., Banks, E., Sivachenko, A., Cibulskis, K., Kernytsky, A., . . . DePristo, M. A. (2010). The genome analysis toolkit: A MapReduce framework for analyzing next-generation DNA sequencing data. *Genome Research, 20*(9), 1297-1303. doi: 10.1101/gr.107524.110

Parejo, M., Wragg, D., Gauthier, L., Vignal, A., Neumann, P., & Neuditschko, M. (2016). Using whole-genome sequence information to foster conservation efforts for the european dark honey bee, *Apis mellifera mellifera*. *Frontiers in Ecology and Evolution, 4*, 140.

Quinlan, A. R., & Hall, I. M. (2010). BEDTools: a flexible suite of utilities for comparing genomic features. *Bioinformatics, 26*(6), 841-842. doi: 10.1093/bioinformatics/btq033

Rimmer, A., Phan, H., Mathieson, I., Iqbal, Z., Twigg, S. R. F., Wilkie, A. O. M., . . . Lunter, G. (2014). Integrating mapping-, assembly- and haplotype-based approaches for calling variants in clinical sequencing applications. *Nature Genetics, 46*(8), 912-918. doi: 10.1038/ng.3036

Van der Auwera, G. A., Carneiro, M. O., Hartl, C., Poplin, R., del Angel, G., Levy-Moonshine, A., . . . DePristo, M. A. (2013). From fastQ data to high-confidence variant calls: The genome analysis toolkit best practices pipeline. *Current Protocols in Bioinformatics*(SUPL.43). doi: 10.1002/0471250953.bi1110s43

Wragg, D., Marti-Marimon, M., Basso, B., Bidanel, J.-P., Labarthe, E., Bouchez, O., . . . Vignal, A. (2016). Whole-genome resequencing of honeybee drones to detect genomic selection in a population managed for royal jelly. *6*, 27168.

Wragg, D., Basso, B., Beguin, M., Canale-Tabet, K., Costa, C., Gregorc, A., ... Vignal, A. (2017). Understanding the French honeybee populations by whole genome sequencing of haploid drones. Manuscript in preparation.

**Supplementary Tables** (available in a separate excel document)

**Table S1.** Number of variable nucleotides on either side of the 250 bp flanking sequences of the fixed SNPs. Marked in bold are the SNPs used for final assay selection.

**Table S2.** Sample origin, coverage statistics and number of variants.

**Table S3.** Genomic information for the 18,272 fixed SNPs (F_ST_=1) between *A. m. iberiensis* and C-lineage honeybees.

**Table S4.** Distribution of SNPs across the 16 chromosomes obtained in the different phases of the assay design.

**Table S5.** Distribution of fixed SNPs by sequence ontology terms.

**Table S6.** Number of fixed SNPs distributed in 1,347 genic regions (±5 kb around coding sequences).

**Table S7.** Significantly enriched gene ontology (GO) terms (P-value < 0.05) for the 1,347 genes harbouring fixed SNPs.

**Table S8.** Primers used for genotyping the 153 highly-informative SNPs in the Agena Bioscience MassARRAY® MALDI-TOF platform.**Table S9.** Performance comparison of reduced (M1-M4) and random (R1-R4) SNP assays in estimating C-lineage proportions (Q-value) in *A. m. iberiensis* holdout and simulated datasets.

**Supplementary Figures**

**Figure S1.** Cross validation (CV) errors for K=1 to 5. CV error is lowest at K=2, suggesting optimal clustering with two ancestral populations.

**Figure S2.** Ancestry proportions (y-axis) from K=2 to 4 clusters. Each individual is represented by a vertical bar and samples are grouped according to subspecies and sample (training and holdout dataset). Each colour represents one cluster and individuals are coloured according to the proportion of the genome that was derived from each cluster. At K=2, the individuals are separated in accordance with the evolutionary lineages M and C. At K=3, the substructure within *A. m. iberiensis* is revealed*,* in line with the geographical cline of this subspecies in Iberia (Chávez-Galarza et al., 2015). At K=3, C-lineage individuals are subdivided into *A. m. carnica* and *A. m. ligustica*.


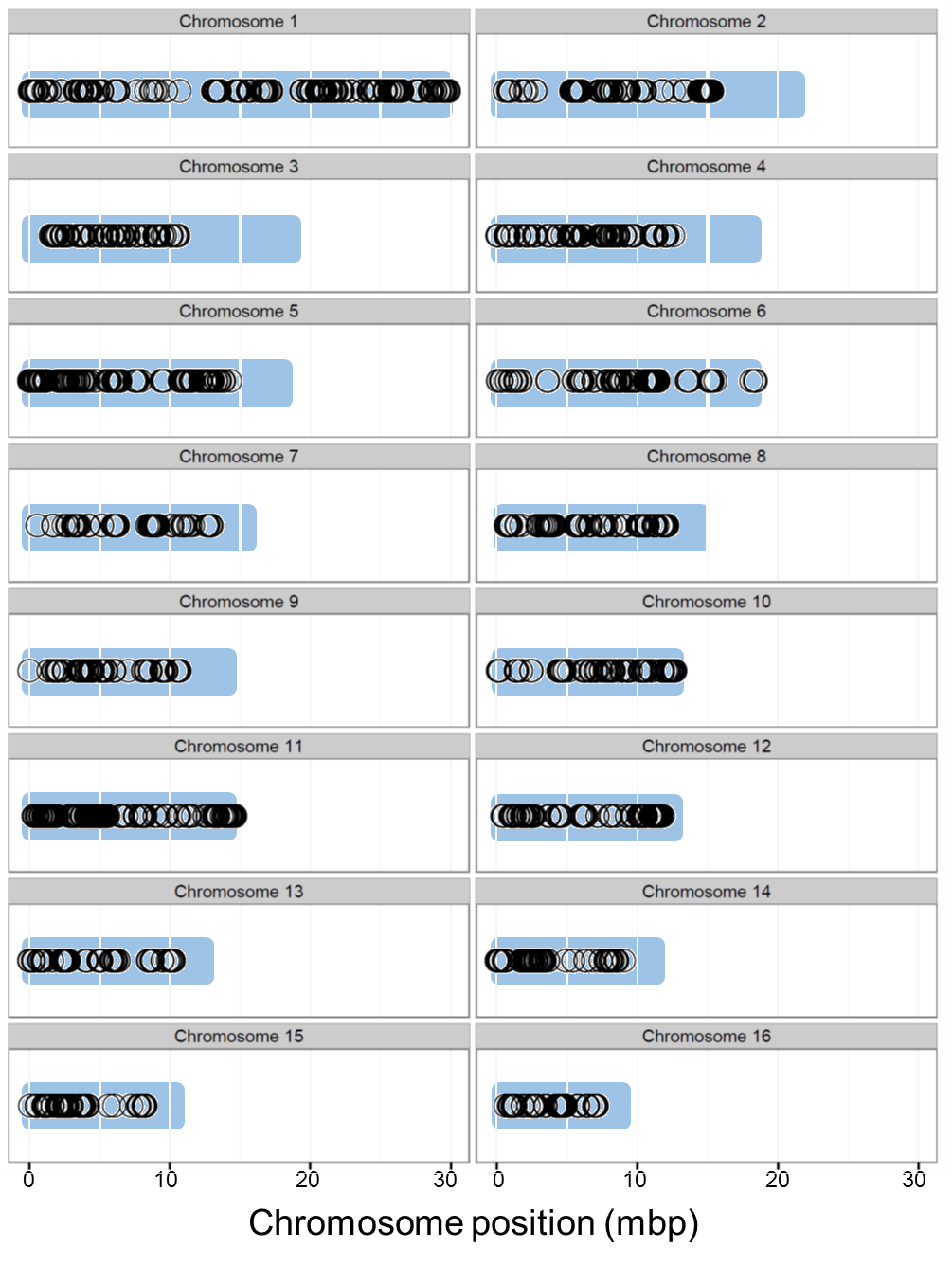


**Figure S3.** Positions of fixed SNPs between *A. m. iberiensis* and C-lineage subspecies (*A. m. carnica* and *A. m. ligustica*) along the 16 honeybee chromosomes (see Table S2 for more detailed information).

**Figure S4.** Linear regression analyses for the ultra-low-density (M1, M2, M3, M4) and random (R1, R2, R3, R4) SNP assays (single or combined) against the whole-genome SNPs.
